# Erythromelalgia Secondary to Anti-Tumor Necrosis Factor (TNF) Alpha Therapy: A Report of Two Cases

**DOI:** 10.7759/cureus.53953

**Published:** 2024-02-10

**Authors:** Fatima Zahra El Rhaoussi, Zineb Boukhal, Fouad Haddad, Mohamed Tahiri, Wafaa Hliwa, Ahmed Bellabah, Badre Wafaa

**Affiliations:** 1 Faculty of Medicine and Pharmacy, Hassan II University, Casablanca, MAR; 2 Department of Gastroenterology and Hepatology, Ibn Rochd University Hospital Center, Casablanca, MAR

**Keywords:** acrosyndrome, tnf alpha inhibitors, infliximab, crohn's disease, erythromelalgia

## Abstract

Erythromelalgia is a rare syndrome with a generally unknown etiology. Whether primary or secondary, this condition is characterized by paroxysmal episodes of erythema, pain, and heat in the extremities. We report two cases of erythromelalgia occurring after the initiation of treatment with infliximab. The first case involves a 38-year-old patient who had been followed since August 2022 for ileocolonic Crohn's disease classified as A2L3B3 according to the Montreal classification, which was resistant to treatment and required infliximab therapy. Two months after the first infusion of infliximab, the patient developed symptoms of erythromelalgia. After ruling out other potential causes through an etiological assessment and conducting a pharmacological investigation, infliximab was considered the most likely cause. Infliximab was discontinued, and symptomatic treatment was initiated, including vascular laser sessions. The patient showed significant clinical improvement. In the second case, a 16-year-old patient with ileocolonic Crohn's disease classified as A1L3B3 according to the Montreal classification was treated with ileocecal resection and received an infusion of infliximab. Sixteen days after the second infusion, she developed clinical symptoms of erythromelalgia. The etiological assessment was inconclusive. Due to a strong suspicion of erythromelalgia secondary to tumor necrosis factor (TNF) alpha inhibitor therapy, infliximab was replaced with ustekinumab. The patient also received symptomatic treatment, and her clinical condition improved, marked by the disappearance of pain.

## Introduction

Erythromelalgia is a rare paroxysmal acrosyndrome characterized by the triad of redness, local warmth, and pain in the extremities. It manifests in paroxysmal episodes triggered by exposure to heat and relieved by the application of cold objects. It affects mostly the lower limbs [1]. Erythromelalgia can be either primary or secondary [1,2]. Secondary forms are often associated with myeloproliferative syndromes, systemic lupus erythematosus, other autoimmune diseases or certain medications, and rarely anti-tumor necrosis factor (TNF) alpha therapy [2]. We present two cases of Crohn's disease having erythromelalgia secondary to infliximab.

## Case presentation

Case 1

A 38-year-old female patient has Crohn's disease in the ileocolonic region classified as A2L3B3 according to the Montreal classification. She was initially treated with corticosteroids and azathioprine with no improvement. Later on, she was put on combination therapy (azathioprine and infliximab). Unfortunately, an intra-abdominal abscess occurred a few weeks later indicating an operation of ileocecal resection.

**Figure 1:**
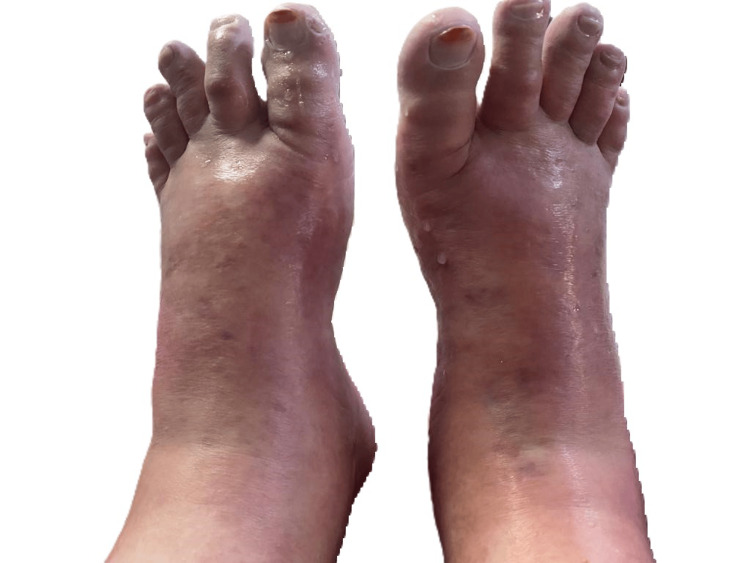
Erythema on the lower limbs, reaching the lower 1/3 of both legs

Postoperatively, two months after the infliximab infusion, she began experiencing paresthesias in her lower limbs and a sensation of heaviness. This was followed by the appearance of erythema on her feet, which was warm and painful, extending to the lower third of both legs (Figure 1). These symptoms were triggered by heat and relieved by cold water.

**Figure 2:**
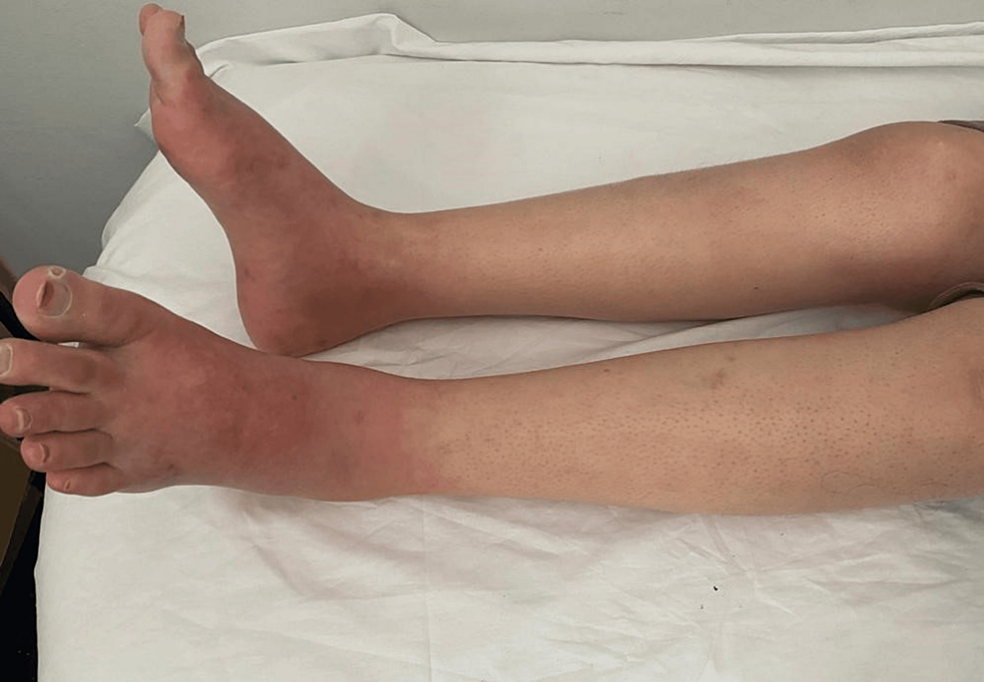
Progress after the second vascular laser session

Laboratory tests showed microcytic hypochromic anemia at 8.7 g/dL (normal range: 12-16 g/dL), negative C-reactive protein (CRP), vitamin B12 level of more than 2000 pg/mL (normal range: 187-883 pg/mL), vitamin B1 level at 229.4 μg/mL (normal range: 26-78 μg/mL), homocysteine level at 10.5 μmol/L (normal range: <15 μmol/L), normal thyroid-stimulating hormone (TSH), and an unremarkable autoimmune profile. Electroneuromyography (ENMG) indicated a sensory-motor polyneuropathy with a predominant sensory component in the lower limbs. Capillaroscopy revealed vascular lesions. After excluding all other causes, infliximab was mostly the probable cause. The patient was treated with antidepressants, antiepileptics, acetylsalicylic acid, and analgesics, in addition to vascular laser sessions. The patient's clinical condition showed significant improvement as the pain and the warmness disappeared (Figure 2).

Case 2

**Figure 3:**
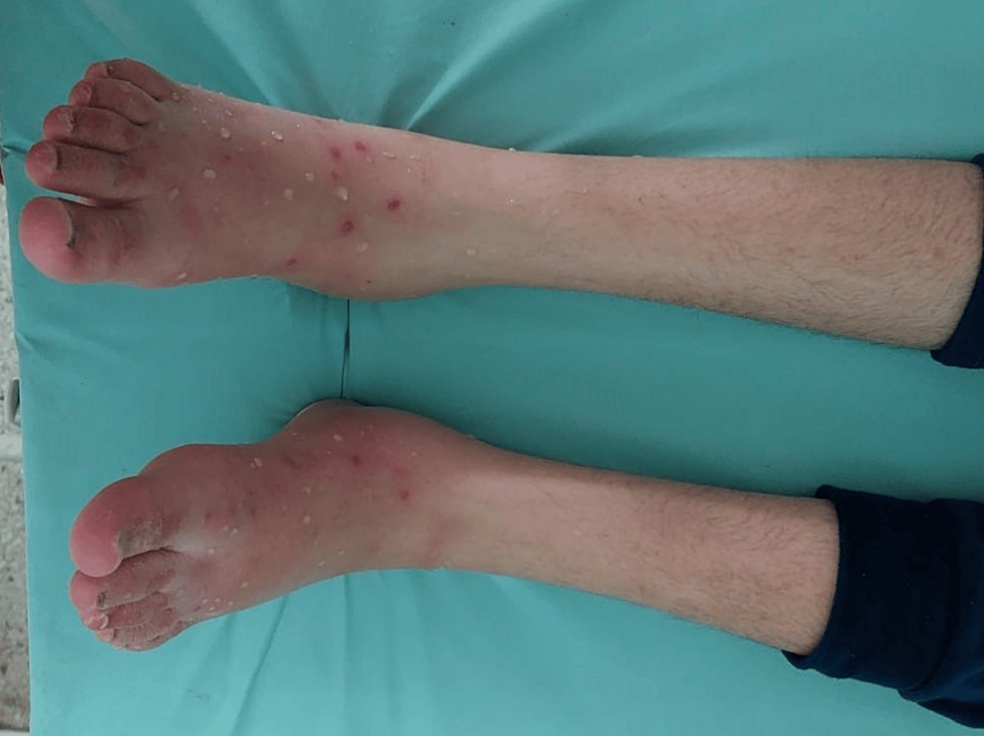
Hot, painful exanthema and infiltrated purpuric lesions on both feet and ankles

**Figure 4:**
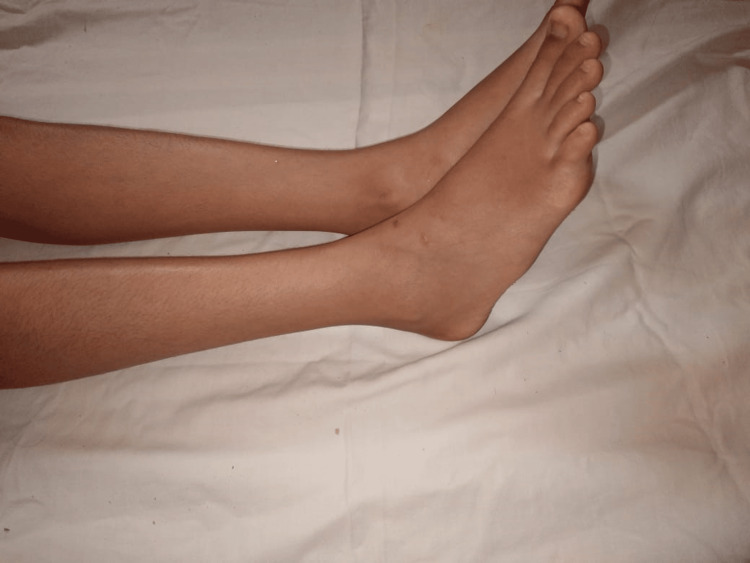
Evolution after stopping infliximab

A 16-year-old female patient, known to have Crohn's disease in the ileocolonic region classified as A1L3B3 according to the Montreal classification, underwent an operation of ileocecal resection. She was put on infliximab postoperatively. After the second session of infliximab infusion, painful rash and purpura appeared on her feet and ankles exacerbated by heat and standing and relieved by cold (Figure 3). Laboratory tests revealed microcytic hypochromic anemia (hemoglobin: 9.4 g/dL), elevated C-reactive protein (CRP) (46 mg/L), vitamin B1 level at 252 mmol/L, vitamin B9 at 2.26 mg/mL, vitamin B12 level of more than 2000 pg/mL, homocysteine level at 6.6 μmol/L, and negative autoimmune markers. Electroneuromyography (ENMG) indicated a predominantly sensory, length-dependent, sensory-motor axonal polyneuropathy. A skin biopsy showed signs of drug-induced leukocytoclastic vasculitis. Erythromelalgia secondary to anti-TNF alpha therapy was highly suspected, and accordingly, infliximab was switched to ustekinumab. The patient also received treatment with antidepressants, antiepileptics, acetylsalicylic acid, and analgesics showing a great outcome (Figure 4).

## Discussion

Erythromelalgia is a rare condition first described by Mitchell in 1878 as a paroxysmal acrosyndrome characterized by burning pain in the fingers and toes accompanied by skin erythema during episodes. Symptoms worsen by heat exposure and are relieved by cold [1]. In 1938, the term "erythromelalgia" was introduced to better account for the increased local heat [2]. Two forms were identified: primary and secondary. Due to its rarity, the true incidence of erythromelalgia is difficult to ascertain. A Norwegian series reported 87 cases (61 females and 26 males), including 48 primary forms (12 with a family history), estimating an incidence of 2/100000 [3]. A population-based study conducted in Minnesota (the USA) between 1976 and 2000 found an average incidence of 1.3/100000, with 1.1/100000 for primary forms and 0.2/100000 for secondary forms [4]. A Swedish retrospective study estimated the incidence at 0.36/100000 based on 27 cases over 10 years [5].

The diagnosis is achieved clinically by stereotypical paroxysmal presentations. During episodes, the extremities, mostly the lower limbs, become red, warm, and painful. However, other body parts may also be affected, such as the upper limbs or ears. Crises are often triggered by physical activity, standing, and heat exposure and alleviated by cold, rest, or the elevation of the affected limbs. It often starts with pruritis in the extremities, which gradually worsens. Pain is often severe and characterized by crushing, burning, or stabbing, which makes the patient immerse his feet in cold water or walk on cold tiles. Symptoms are more severe and frequent in summer and worsen at bedtime. The patient prefers sleeping with uncovered feet. The duration of episodes varies from minutes to hours or even days [6]. Erythromelalgia does not lead to trophic changes. However, wounds and pseudo-ischemic lesions secondary to prolonged cold-water baths have been reported. Since the patients are rarely seen during an episode, the diagnosis initially relies on a thorough medical history, and it is not always easy to determine if there is local warmness during the crises [6,7].

As erythromelalgia is suspected based on clinical presentation, diagnostic criteria have been proposed by different teams to consider the diagnosis exclusively in patients whose pain is accompanied by obvious vasodilation, manifested as redness and local warmness [8,9].

The differential diagnosis between primary erythromelalgia and secondary erythromelalgia is based on a careful medical history and a search for medications such as calcium channel blockers and anti-TNF drugs. Complete blood count is essential in searching for myeloproliferative syndromes. Moreover, immunological marker tests of rheumatoid arthritis, cryoglobulinemia, and cutaneous vasculitis should be done [10]. The diagnosis of erythromelalgia secondary to infliximab in our patients was made because of the absence of all other causes.

The therapeutic management of erythromelalgia remains challenging. In patients with secondary erythromelalgia, treating the underlying disease often helps in reducing the symptoms [11]. There is currently no treatment that offers complete efficacy for all patients. The commonly recommended treatments for neuropathic pain are applied [12,13]. The main treatment includes antiepileptics and antidepressants. The use of topical products or botulinum toxins should not be overlooked [14,15]. The first-line recommended treatment is pregabalin, gabapentin (antiepileptics), duloxetine, or amitriptyline (antidepressants) [16]. Tramadol should be additionally used for acute painful episodes. In case of failure or partial response, dose escalation or replacement or a combination of drugs may be considered. Local treatments (lidocaine patches and capsaicin) can be useful for localized pain and can be used alone or in combination with systemic treatments [17]. The combination of antidepressant, antiepileptic, acetylsalicylic acid, and local anesthetics showed great outcomes in our patients.

The disease's course is variable and associated with significant morbidity, including the possibility of death, as illustrated by a retrospective study of 32 cases: the follow-up of 15 cases over an average of 9.1 years showed disease stability in five patients, improvement in four, resolution in two, and worsening in one. Three patients died (including one by suicide) [18]. A significant clinical improvement was observed in both of our patients.

## Conclusions

Secondary erythromelalgia caused by TNF alpha inhibitors is a rare clinical diagnosis that significantly impairs the quality of life. Literature reviews encourage discussing, on a case-by-case basis, the discontinuation of treatment and the initiation of symptomatic treatment.
